# Prohexadione-calcium alleviates the leaf and root damage caused by salt stress in rice (*Oryza sativa L*.) at the tillering stage

**DOI:** 10.1371/journal.pone.0279192

**Published:** 2023-03-17

**Authors:** Rongjun Zhang, Dianfeng Zheng, Naijie Feng, Quan-Sheng Qiu, Hang Zhou, Fengyan Meng, Xixin Huang, Anqi Huang, Yixiang Li

**Affiliations:** 1 College of Coastal Agricultural Sciences, Guangdong Ocean University, Zhanjiang, China; 2 National Saline-tolerant Rice Technology Innovation Center, South China, Zhanjiang, China; 3 Shenzhen Institute of Guangdong Ocean University, Shenzhen, China; 4 MOE Key Laboratory of Cell Activities and Stress Adaptations, School of Life Sciences, Lanzhou University, Lanzhou, Gansu, China; University of Alberta, CANADA

## Abstract

Salt stress, as a principal abiotic stress, harms the growth and metabolism of rice, thus affecting its yield and quality. The tillering stage is the key growth period that controls rice yield. Prohexadione-calcium (Pro-Ca) can increase the lodging resistance of plants by reducing plant height, but its effects on rice leaves and roots at the tillering stage under salt stress are still unclear. This study aimed to evaluate the ability of foliar spraying of Pro-Ca to regulate growth quality at the rice tillering stage under salt stress. The results showed that salt stress reduced the tillering ability of the rice and the antioxidant enzyme activity in the roots. Salt stress also reduced the net photosynthetic rate (Pn), stomatal conductance (Gs) and intercellular CO_2_ concentration (Ci) of the rice leaves and increased the contents of osmotic regulatory substances in the leaves and roots. The application of exogenous Pro-Ca onto the leaves increased the tiller number of the rice under salt stress and significantly increased the photosynthetic capacity of the leaves. Additionally, it increased the activities of antioxidant enzymes and the AsA content. The contents of an osmotic regulation substance, malondialdehyde (MDA), and H_2_O_2_ in the leaves and roots also decreased. These results suggested that Pro-Ca can increase the tillering ability, photosynthetic capacity, osmotic adjustment substance content levels and antioxidant enzyme activity levels in rice and reduce membrane lipid peroxidation, thus improving the salt tolerance of rice at the tillering stage.

## Introduction

In recent years, the global saline-alkali cultivated land has been growing rapidly due to natural and human factors [1]. The effects of antioxidation and lipid peroxidation caused by salt stress in the leaves and roots of salt-tolerant and salt-sensitive maize genotypes have been reported. The saline-alkali cultivated land in China is also expanding, accounting for approximately 1.03% of the total land area [2]. As a major abiotic stress, salt stress seriously affects the growth and yield of crops. This also restricts the development of agricultural productivity [3].

Rice is the leading food for more than half of the world’s population, and more than 90% of rice is grown in Asia [4]. The yield and quality of rice are highly sensitive to salt stress, and different rice varieties and growth periods have different responses to salt stress [5]. The tillering stage is a critical period as it determines the rice plant structure, panicle number and final yield and a reasonable tiller number and tillering angle are the critical factors that determine the quality and yield of rice [6]. As a gramineous plant, new tillers of rice and other crops occur on the main stem, and these tillers have independent roots, which help ensure the survival of the plant under different conditions [7]. Salt stress can significantly inhibit rice growth, and roots are the first part for plants to perceive steady-state water nutrition dynamics in the soil [8]. Salt reduces water absorption in plants through osmosis and produces osmotic stress [9]. Subsequently, ion transporter-related transporters play a role in plant roots, transporting and accumulating Na^+^ and Cl^-^, causing ion toxicity in the cytoplasmic ectoplasm or cytoplasm via the symplastic pathway [10]. Excessive accumulation of Na^+^ in plant cells hinders the transport of other ions, which leads to ion imbalance, disrupts photosynthetic capacity, causes oxidative damage, and damages cell membranes, which further affects plant growth, including the reduction of leaves, photosynthetic capacity, and the water use efficiency of leaves [11–13]. One study showed that moderate salt stress (EC 5 dS/m) inhibited rice spikelet development, decreased tiller number, panicle number, and spikelet number, and seriously affected rice yield [5]. Therefore, to meet the food demand of the growing global population, it is essential to improve the salt tolerance of crops and reduce the damage of salt stress on rice yields [14].

Plant growth regulators are synthetic or extracted organic compounds used to regulate plant growth and development. Exogenous application of different plant growth regulators is widely used in crops because of their advantages of no secondary pollution and their significant effects in small amounts. Plant growth regulators, directly and indirectly, participate in the regulation of crop tolerance mechanisms under stress by regulating photosynthesis and antioxidant metabolism [15]. Foliar spraying of blueberry anthocyanins on leaves has been shown to form chelating compounds with Cd ions to fix Cd in rice leaves, effectively alleviating the oxidative damage of Cd [16]. It has also been documented that spraying indole-3-acetic acid (IAA), gibberellin GA_3_ (GA_3_), 6-benzaminopurine (6-BA) and 24-epbrassinolide (EBL) on the leaf surfaces of Brassica juncea improved the tolerance and repair efficiency to Cd and U contamination [17]. Prohexadione-calcium (Pro-Ca) is hypothesized to be competitive with 2-oxoglutarate and cause the inhibition of the 2-oxoglutarate-dependent dioxygenases involved in gibberellin biosynthesis and thus regulate the late stage of GA formation by interfering with 3β-hydroxylation [18, 19]. This can reduce plant height, increase stem base width and improve the lodging resistance of crops. It has been proven that Pro-Ca has specific regulatory effects on rice, apples, strawberries and various vegetable and cereal crops [18, 20, 21]. Pro-Ca can also effectively alleviate the damage to plants caused by salt stress in many crops. Pro-Ca can increase the contents of amino acids, proline, photosynthetic pigments, total carbohydrates and total soluble sugar in broad bean seedlings under salt stress [22]. Feng et al. [23] showed that foliar spraying of exogenous Pro-Ca effectively protected soybean seedlings from saline-alkali stress by increasing the chlorophyll content, accumulation of Pn, Fv/F0, Fv/Fm, ETR, AsA, soluble sugar, and proline in soybean leaves, and enhanced antioxidant defences. Aghdam [24] indicated that Pro-Ca reduced the damage caused by chilling stress in tomatoes by inhibiting PLD and LOX activities and reducing membrane damage.

However, there are few reports about the alleviating effect of Pro-Ca on the morphology and physiological characteristics of the aboveground and belowground parts of rice at the tillering stage under salt stress. This paper compares the morphological indices, photosynthetic indices, antioxidant indices, and membrane damage indices of the aboveground and belowground parts of rice after spraying Pro-Ca on the leaves of rice at the tillering stage under salt stress to explore the harmful effects of salt stress on rice at the tillering stage and the regulatory effect of Pro-Ca. To date, there are few reports about the application of Pro-Ca in rice by leaf spraying under salt stress, especially in the tillering stage of rice. This study provided some insights into the damage of salt stress on the phenotype and physiological mechanism of two rice varieties at the tillering stage and the impacts of foliar spraying of Pro-Ca. In particular, morphological indices, photosynthetic characteristics, antioxidant indices, and membrane damage indices of the aboveground and belowground plant parts were compared.

## Materials and methods

### Material and reagent collection

Seeds of two rice (Oryza sativa L.) varieties, one inbred rice, Huanghuazhan (HHZ), was obtained from Longping Seed Co., Ltd., Hunan, China, and the other hybrid rice variety, Xiangliangyou 900 (X900), was obtained from Nianfeng Seed Technology Co., Ltd., Hunan, China. The chemical reagent’s original solution was 5% Pro-Ca, provided by the College of Coastal Agricultural Sciences, Guangdong Ocean University. The regulator concentration was 100 mg∙L^-1^.

### Experimental design

The experiment was conducted in the solar greenhouse of Guangdong Ocean University in 2021. The seeds with a consistent growth state were surface sterilized with 2.5% sodium hypochlorite (NaClO) for 15 minutes, rinsed with distilled water, soaked in distilled water at 30°C for 24 hours and then germinated with distilled water in the dark for 24 hours at 30°C. The germinated seeds were evenly spread on the seedling raising tray (28 × 58 cm in size) with approximately 5~8 seeds in each hole and then covered with soil. The soil used for the seeds was raising was a mixture of latosol and nutrient soil at a ratio of 3:1 (Latosol, equivalent to Ferralsols in WRB classification, is an Fe-Al soil with an apoplastic layer, a dark reddish-brown surface layer, and a brick-red Fe-Al residual B layer under a tropical rainforest or monsoon forest. It is gray-brown in color, more than 15–30 cm thick, and has 8–10% organic matter content. However, mineralization is also strong, with large dispersion, slight flocculation and unstable aggregates). Plastic pots with a diameter × bottom diameter × height of 19 × 15 × 18 cm were used as the experimental pots, and each plastic pot was filled with 3 kg of latosol. Seven days before transplanting the rice seedlings, 1 L of water was added to each pot to soak and stir the soil, and when the water surface was stable, the heights of the water layer were marked. Then, water was added regularly to maintain the marked water level. Fertilization was applied 1 day before transplanting. When the seedlings grew to three leaves and one heart in the seedling tray, they were transplanted. The transplanting depth was approximately 1.5 cm, and each pot had three holes, each hole had one plant, and the distance between each hole was 10 cm. Throughout the experiment, the leaf age was marked every five days.

After turning green, the plants were foliar sprayed with Pro-Ca on the leaves before tillering, and the spraying time was 6:00–8:00 p.m. on a sunny day. The front and back of each leaf were sprayed evenly to moisten, but not drip, to ensure normal absorption. After 24 hours of the Pro-Ca treatment, the soil and salt were treated with salt at a ratio of 1000:3 (W/W), and the salt was added twice. Fourteen days after the salt treatment, the conductivity of the soil measured by a soil salinity metre (Shunkeda TR-6D) was 5.660 dS/m. The water layers were kept at the marked heights from the beginning to the end of the experiment. There were four treatments for each variety: (i) CK (distilled water + 0% NaCl), (ii) S (distilled water + 0.3% NaCl), (iii) Pro-Ca (100 mg∙L^-1^ Pro-Ca + 0% NaCl), and (iv) Pro-Ca+S (100 mg∙L^-1^ Pro-Ca + 0.3% NaCl). On the 14^th^ day after the salt treatment, samples were taken to determine the related morphological and physiological indices.

### Measurement of morphological and photosynthetic indices

Samples were taken on the 14^th^ day after the salt stress treatment to measure morphological indices. The branch with more than two leaves was counted as one tiller, and the number of tillers was calculated based on this standard. The morphology of the fifth leaf axil and its length were measured by removing the fifth leaf sheath of the main stem. The plant height, root length, and stem base width were measured directly by a ruler and a Vernier calliper, and the leaf area of the functional leaves from each main stem was measured by a leaf area metre YX-1241. The fresh weights of the shoots and roots were measured by an electronic balance, placed in an oven at 105°C for 30 min, and then dried to a constant weight at 75°C. Then, the dry weights of the shoots and roots were measured by an electronic balance. The root-to-shoot ratio is the ratio of the belowground dry weight to the aboveground dry weight.

On the 14^th^ day after the NaCl stress, a portable chlorophyll SPAD-502 (Konica Minolta, Japan) was used to determine the SPAD value of the penultimate leaf on the main stem. The net photosynthetic rate (Pn), transpiration rate (Tr), stomatal conductance (Gs), and intercellular CO_2_ concentration (Ci) of the leaves (of the penultimate leaf on the main stem) were measured at 9:00–11:30 a.m. with a portable photosynthetic apparatus (LI-COR, Inc. USA). During the measurement, the relative humidity was kept at 70%. For the calculation methods of the water use efficiency (WUE) and apparent mesophyll conductance (AMC) refer to Chen et al. [25]. The WUE is the ratio of Pn to Tr (Pn/Tr). AMC is the ratio of Pn to Ci (Pn/Ci).

### Measurement of the membrane damage index

Electrolyte leakage was measured according to the method described by Ahmad et al. [26]. The fresh leaves (0.1 f of the functional leaves) were soaked in 10 ml deionized water at room temperature for 24 hours, and then the conductivity (R1) was measured by a conductivity metre. The samples were boiled in boiling water for 30 minutes, and the conductivity (R2) was measured after cooling. The blade electrolyte leakage (El) was calculated according to the formula: El = R1/R2 × 100%.

The malondialdehyde (MDA) content was determined by the TBA method [27]. The functional leaves (0.5 g) were ground in liquid nitrogen, and then 10 ml phosphate buffer (0.05 mM PBS, pH 7.8) was added, ground into homogenate, and centrifuged at 10,000 × g and 4°C for 10 min. The supernatant (1 ml) was mixed with 2 ml of 0.6% TBA (thiobarbituric acid) in a centrifuge tube. The mixture was boiled in a boiling water bath for 15 minutes and then centrifuged at 10,000 × g and 25°C for 10 min. The absorbance of the supernatant was measured at 450 nm, 532 nm, and 600 nm using a spectrophotometer (GENESYS 180 UV-Vis, Thermo Sci).

For the determination of the H_2_O_2_ content, referring to the method of Jessup et al. [28], 0.5 g of the leaf samples were taken, 5 ml of 0.1% TCA solution was added, ground in liquid nitrogen, and centrifuged at 10000 × g for 10 min. Then, 0.5 ml of the supernatant was added to 0.5 ml of a 10 mM PBS buffer and 1 ml of KI solution and reacted in the dark at 28°C for 1 h, The absorbance was measured at 390 nm by a spectrophotometer (GENESYS 180).

### Measurement of the antioxidant system

On the 14^th^ day after the NaCl stress, the functional leaves of the rice main stem were quickly frozen in liquid nitrogen and then stored at -80°C. The leaves (0.5 g) were ground in liquid nitrogen, 10 ml of a precooled phosphate buffer (0.05 mM PBS, pH 7.8) was added, ground into homogenate, and centrifuged at 4°C for 20 min at 6000 × g. And the activity levels of superoxide dismutase (SOD), catalase (CAT), peroxidase (POD), and ascorbate peroxidase (APX) within the supernatant were measured. The activity of SOD was determined by the NBT nitrotetrazolium blue method. Then, 0.1 ml of an enzyme solution was added to 2.9 ml of a reaction mixture (2.61 ml met+0.097 ml EDTA-Na_2_+0.097 ml NBT+0.097 ml riboflavin), and the mixture was centrifuged at 4000 × g for 20 min at 25°C. The absorbance was measured at 560 nm using a spectrophotometer (GENESYS 180), and the total activity levels of SOD were calculated by the method of Giannopolitis and Ries [29]. Then, 0.1 ml of an enzyme solution was mixed with 2.9 ml of a reaction solution (PBS pH 7.0 + 30% H_2_O_2_), and the absorbance at 240 nm was measured and recorded every 30 seconds four times with a spectrophotometer (GENESYS 180). The activity of CAT was calculated according to the method provided by Aebi [30]. Next,30 ml of a reaction solution (PBS pH 6.0 + guaiacol) was mixed with 40 microlitres of an enzyme solution. The absorbance was recorded every 30 seconds four times, and the dynamic absorbance was measured at 470 nm using a spectrophotometer (GENESYS 180). The POD activity was calculated according to the method reported by Klapheck et al. [31]. Then, 0.1 ml of an enzyme solution was mixed with a reaction solution (2.6 ml EDTA-Na_2_+0.15 ml AsA+0.15 ml H_2_O_2_), and the dynamic absorbance at 290 nm was measured at 20°C every 30 seconds four times with a spectrophotometer (GENESYS 180). The APX activity level was calculated according to the method described by Nakano and Asada [32].

For the determination of AsA, 0.5 g of the plant leaves was taken, 10 ml of a 5% TCA solution was added, ground at 4°C, and centrifuged at 20000 r/min for 15 min. One millilitre of the sample extract was placed in a test tube, 1.0 ml of 5% TCA and 1.0 ml of ethanol were added, and the mixture was shaken well. Then, 0.5 ml of 0.4% H_3_PO_4_-ethanol, 1.0 ml of 0.5% BP-ethanol, and 0.5 ml of 0.03% FeCl_3_-ethanol were added. The solution was reacted at 30°C for 90 min, OD534 was determined using a spectrophotometer (GENESYS 180), and AsA content was calculated according to the standard curve [33].

GSH was measured according to Ellman [34], 1.0 g of the plant leaves was taken, 5 ml of 5% metaphosphoric acid solution was added, ground at 4°C, and centrifuged at 20000 × g for 15 min. Then, 0.2 ml of the supernatant was added to 2.6 ml of a NaH_2_PO_4_ solution (pH 7.7) and 0.2 ml of a DTNB solution, the temperature was maintained at 30°C for 10 min, and the absorbance was measured at 412 nm by a spectrophotometer (GENESYS 180).

### Measurement of osmotic adjustment substances

For the determination of the soluble protein content, referring to the method of Bradford [35], 0.5 g of sample was weighed in a precooled mortar, 10 ml of a 0.05 mol/L precooled phosphoric acid buffer (pH 7.8) was added three times, ground into homogenate in an ice bath, transferred into a centrifuge tube, and centrifuged at 12000 × g at 4°C for 2 min, and the supernatant was determined as the crude protein extract. One millilitre of an enzyme solution was added to 5 ml of a Coomassie brilliant blue solution and shaken evenly.

For the determination of proline content, 0.3 g of the fresh plant tissue samples were weighed, a proper amount of 80% ethanol and a small amount of quartz sand were added, ground into a homogenate in a mortar, fixed to 10 ml with 80% ethanol, and then extracted in a water bath at 80°C for 20 min. A spoonful of artificial zeolite (to remove interfering amino acids) and a small amount of activated carbon were added to the extract, shaken vigorously for 5 min, and filtered for later use. A total of 2 ml of proline extract was added to a calibrated test tube, 4 ml of 1.25% acid ninhydrin was added, and the tube was heated in a boiling water bath for 30 min. Then, the absorbance of each sample was measured at 508 nm by a spectrophotometer (GENESYS 180) after cooling [36].

### Statistical analysis

The data are expressed as the mean standard error (SEM) of the mean value. The statistical significance was determined by one-way ANOVA and Duncan’s comparison test (*P* < 0.05). The digital charts were compiled by using Origin 2021. SPSS 25.0 and Origin 2021 were used for the correlation analysis.

## Result

### Effects of exogenous prohexadione-calcium (Pro-Ca) on morphological indices of rice leaves and roots at the tillering stage under salt stress

**Fig 1:**
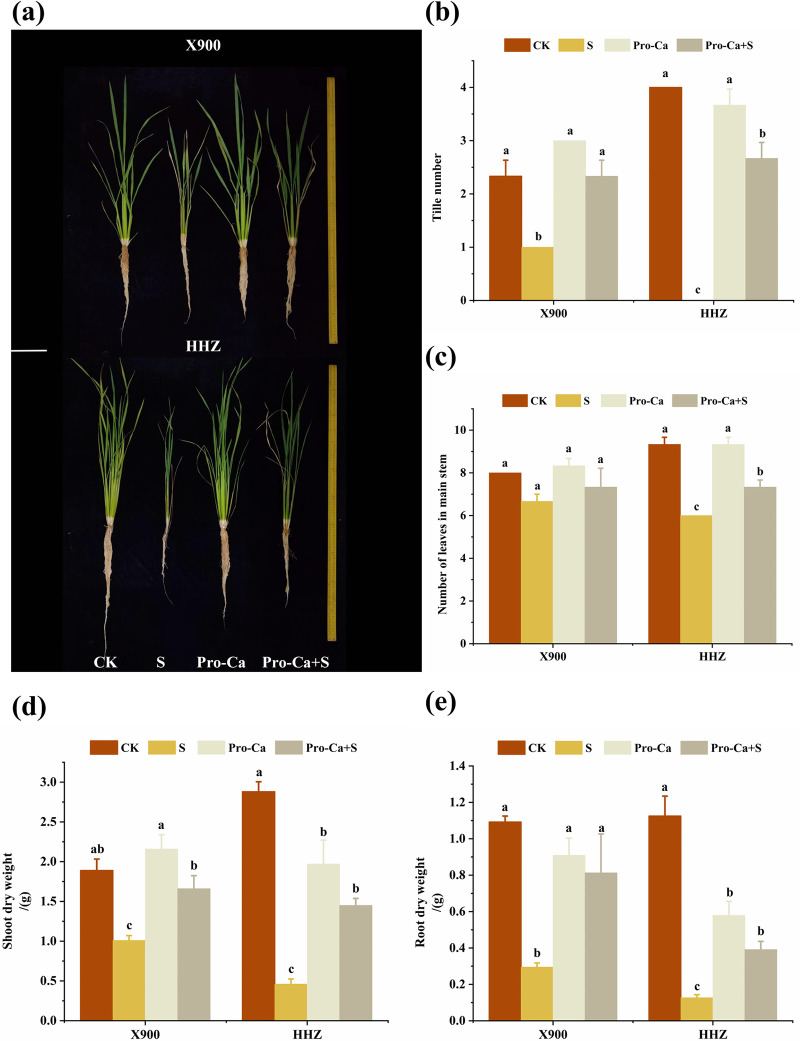
Alleviating effect of exogenous prohexadione-calcium (Pro-Ca) on tillering ability of rice under salt stress. The morphology (a), tiller number (b), number of leaves in the main stem (c), and shoot and root dry weight (d and e) of the X900 and HHZ varieties under different treatments. Abbreviations: CK (water spraying + 0% NaCl), S (water spraying + 0.3% NaCl), Pro-Ca (100 mg∙L^-1^ Pro-Ca leaf spraying + 0.3% NaCl), and Pro-Ca + S (100 mg∙L^-1^ Pro-Ca leaf spraying + 0.3% NaCl). The numerical value represents the mean ± SE (*n =* 3), and different lowercase letters indicate significant differences according to Duncan’s test (*p <* 0.05).

**Fig 2:**
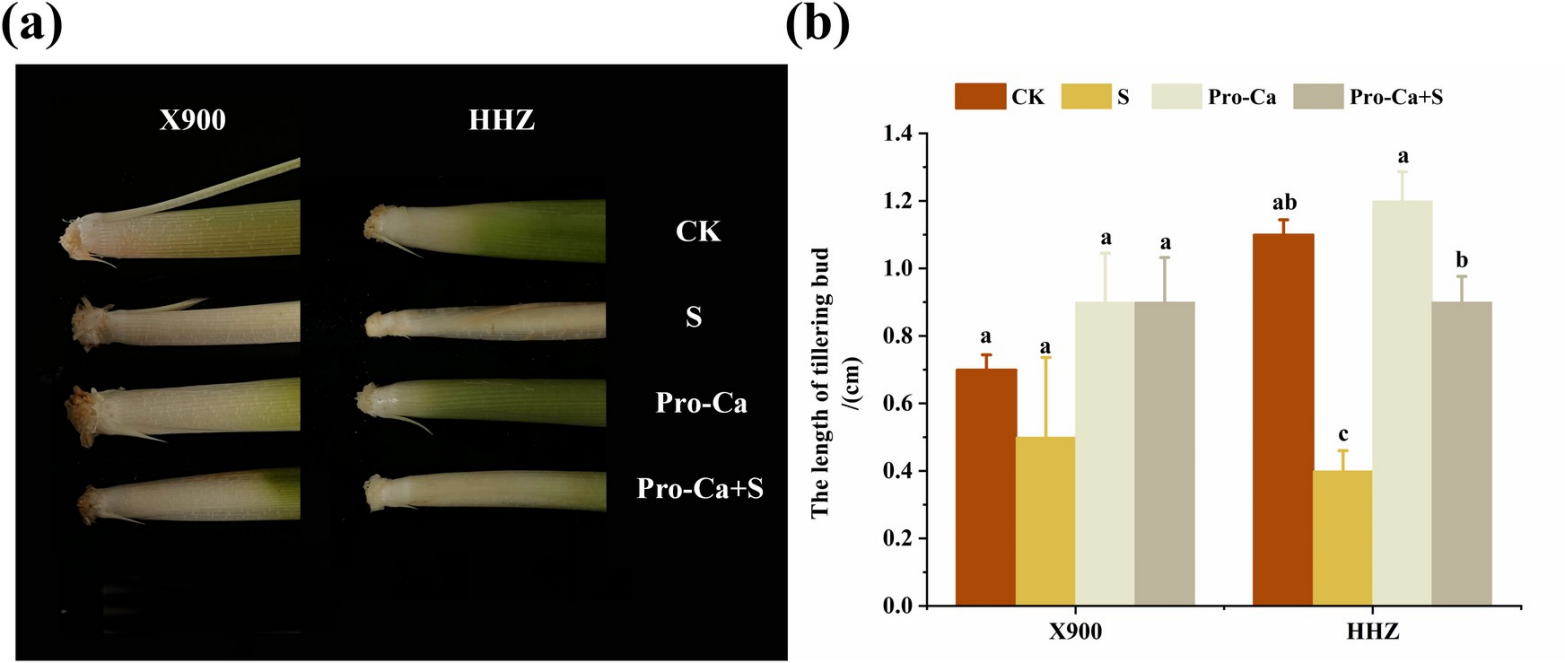
Effect of exogenous prohexadione-calcium (Pro-Ca) on the length of the axillary buds of the fifth leaf of rice under salt stress. Abbreviations: CK (water spraying + 0% NaCl), S (water spraying + 0.3% NaCl), Pro-Ca (100 mg∙L^-1^ Pro-Ca leaf spraying + 0.3% NaCl), and Pro-Ca + S (100 mg∙L^-1^ Pro-Ca leaf spraying + 0.3% NaCl). The numerical value represents the mean ± SE (*n =* 3), and different lowercase letters indicate significant differences according to Duncan’s test (*p <* 0.05).

The results showed that salt stress significantly inhibited the growth of Huanghuazhan (HHZ) and Xiangliangyou900 (X900) at the tillering stage. The tillering number of X900 decreased by 57.14% under salt stress, and no tillering occurred in HHZ under salt stress. The main stems and leaves also decreased by 16.67% and 35.71%, respectively (Fig 1A–1C). Salt stress also significantly inhibited the growth of the tillering buds of the two rice varieties (Fig 2). Compared with the control, the root length, stem base width, and leaf area of the two varieties also decreased by 29.92% and 54.27%, 29.31% and 66.79%, and 58.43% and 30.70%, respectively, under salt stress (Table 1). Salt stress also inhibited the biomass of the two rice varieties. In the S treatment, the aboveground and belowground fresh weights and aboveground and belowground dry weights of X900 and HHZ decreased by 42.98% and 69.92%, 44.24% and 86.16%, 46.79% and 84.01%, and 72.98% and 88.71%, respectively, relative to the CK treatment group (Table 2 and Fig 1D and 1E). The root-shoot ratio of the two rice varieties also decreased by 48.57% and 28.81% under NaCl stress, but the difference was not significant (Table 2). The above results demonstrate that salt stress strongly inhibited both rice varieties, and the damage to the HHZ variety under salt stress was more severe than that of the X900 variety.

**Table 1:** Effects of exogenous prohexadione-calcium (Pro-Ca) on plant height, root length, stem base width, and leaf area at the tillering stage under salt stress.

Varieties	treatments	Plant height /(cm)	Root length /(cm)	Stem base width /(mm)	Leaf area /(mm^2^)
X900	CK	72.87±2.19a	35.43±1.62a	26.3±2.5a	5348.9±224.4a
	S	47.63±1.39c	24.83±2.28b	18.6±0.4b	2223.3±418.1c
	Pro-Ca	64.27±3.07b	31.80±2.06ab	27.4±0.8a	5701.1±336.5a
	Pro-Ca+S	53.73±1.66c	31.90±2.31ab	22.3±2.5ab	3876.8±57.6b
HHZ	CK	69.47±0.29a	44.90±3.12a	26.7±0.8a	3621.1±896.8a
	S	54.43±2.35b	20.53±3.45c	8.9±0.3c	2509.6±139.9a
	Pro-Ca	65.30±1.91a	35.40±2.47b	25.2±2.2ab	3847.6±291.2a
	Pro-Ca+S	59.27±1.32b	30.77±1.62b	21.3±0.9b	3943.7±439.6a

Values represent the mean ± SE (*n  =*  3), and different lowercase letters indicate significant differences according to Duncan’s test.

**Table 2:** Effects of exogenous prohexadione-calcium (Pro-Ca) on the root-to-shoot ratio, plant mass-height ratio, shoot fresh weight, and root fresh weight at the tillering stage under salt stress.

Varieties	treatments	Root to shoot ratio	Shoot fresh weight /(g)	Root fresh weight /(g)
X900	CK	0.58±0.06a	10.2567±0.5880ab	6.7022±0.8940ab
	S	0.30±0.04a	5.8481±0.4475c	3.7371±0.4893b
	Pro-Ca	0.42±0.04a	11.6324±0.7687a	7.1243±1.1836a
	Pro-Ca+S	0.50±0.15a	9.3541±0.5296b	5.8021±1.1271ab
HHZ	CK	0.39±0.05a	12.4722±0.2317a	7.8273±0.86651a
	S	0.28±0.03a	3.7508±0.6873c	1.0831±0.18006c
	Pro-Ca	0.30±0.02a	9.1614±1.4250b	4.144±0.64684b
	Pro-Ca+S	0.28±0.04a	8.5918±0.6314b	4.1068±0.50988b

Values represent the mean ± SE (*n  =*  3), and different lowercase letters indicate significant differences according to Duncan’s test.

The results of the Pro-Ca+S treatment showed that exogenous Pro-Ca helped alleviate the damage to rice morphology caused by NaCl stress. After spraying Pro-Ca under NaCl stress, the tillering number and the number of main stems and leaves of the X900 and HHZ plants were relieved by 100% and 66.67%, 50%, and 40%, respectively (Fig 1A–1C). Spraying exogenous Pro-Ca also significantly alleviated the salt stress inhibition on the rice fifth tiller bud (Fig 2). The root length, stem base width, and leaf area of the two varieties were also significantly improved, and the related indices of the X900 and HHZ plants were relieved by 66.67% and 42%, 58.05% and 69.53%, 52.9% and 129.02%, respectively (Table 1). The spraying of Pro-Ca significantly increased the biomass of the two rice varieties, and the fresh weight and dry weight of the X900 and HHZ plants were relieved by 79.63% and 55.51%, 69.64% and 44.83, 74.28% and 41.10%, 64.96% and 26.49%, respectively, compared with the control salt stress treatment group (Table 2 and Fig 1D and 1E). At the same time, the plant mass-height ratio of the X900 plants was improved by 198.51%, while the HHZ plants were improved by 44.33%, with a significant difference.

### Effects of exogenous prohexadione-calcium (Pro-Ca) on the photosynthetic capacity of rice leaves at the tillering stage under salt stress

**Fig 3:**
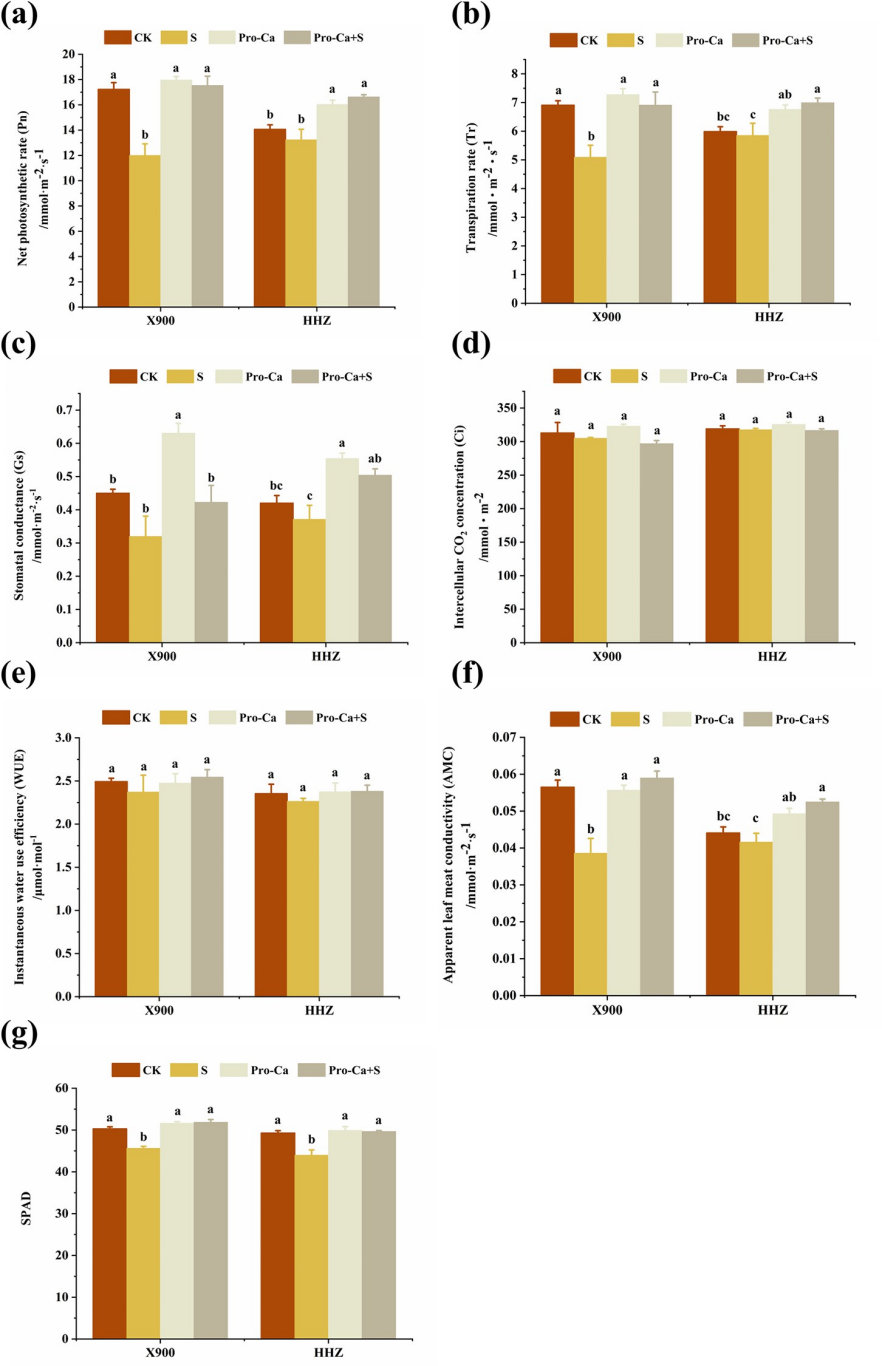
Alleviating effect of exogenous prohexadione-calcium (Pro-Ca) on the photosynthetic capacity of rice under salt stress. a: Net photosynthetic rate (Pn) in the X900 (left) and HHZ (right) plants; b: transpiration rate (Tr) in the X900 (left) and HHZ (right) plants; c: stomatal conductance (Gs) in the X900 (left) and HHZ (right) plants; d: intercellular CO_2_ concentration (Ci) in the X900 (left) and HHZ (right) plants; e: instantaneous water use efficiency (WUE) in the X900 (left) and HHZ (right) plants; f: apparent leaf meat conductivity (AMC) in the X900 (left) and HHZ (right) plants; g: SPAD in the X900 (left) and HHZ (right) plants. Abbreviations: CK (water spraying + 0% NaCl), S (water spraying + 0.3% NaCl), Pro-Ca (100 mg∙L^-1^ Pro-Ca leaf spraying + 0.3% NaCl), and Pro-Ca+S (100 mg∙L-1 Pro-Ca leaf spraying + 0.3% NaCl). The numerical value represents the mean ± SE (*n =* 3), and different lowercase letters indicate significant differences according to Duncan’s test (*p <* 0.05).

Fig 3 shows that salt stress had a significant impact on the photosynthetic indices of the two varieties of rice, among which the Pn, Gs, Tr, AMC, Ci, and WUE of the medium salt-tolerant hybrid rice X900 decreased by 30.37%, 28.96%, 26.29%, 31.68%, 2.56%, and 4.86%, respectively, but not significantly under salt stress. Compared with the control, Pn, Gs, and Ci in HHZ decreased by 5.92%, 11.60%, and 0.42%, respectively, without significance (Fig 3A–3D). Tr, WUE, and AMC decreased by 2.28%, 3.81%, and 5.67%, respectively (Fig 3B, 3E, and 3F). In addition, the SPAD value of the two rice varieties decreased by 9.28% and 10.75%, respectively (Fig 3G).

The results of the Pro-Ca+S treatment showed that exogenous Pro-Ca significantly enhanced the photosynthetic capacity of the two rice varieties, among which the Pn of the X900 and HHZ plants recovered by 105.73% and 407.97%, respectively, and the Gs was improved by 79.05% and 273.10%, respectively. These results indicated that Pro-Ca significantly alleviated the Tr, WUE, AMC, and SPAD values of the two varieties. Meanwhile, exogenous spraying of Pro-Ca reduced the Ci of the rice (Fig 3).

### Effects of exogenous prohexadione-calcium (Pro-Ca) on the membrane damage index and H_2_O_2_ content of rice leaves and roots at the tillering stage under salt stress

**Fig 4:**
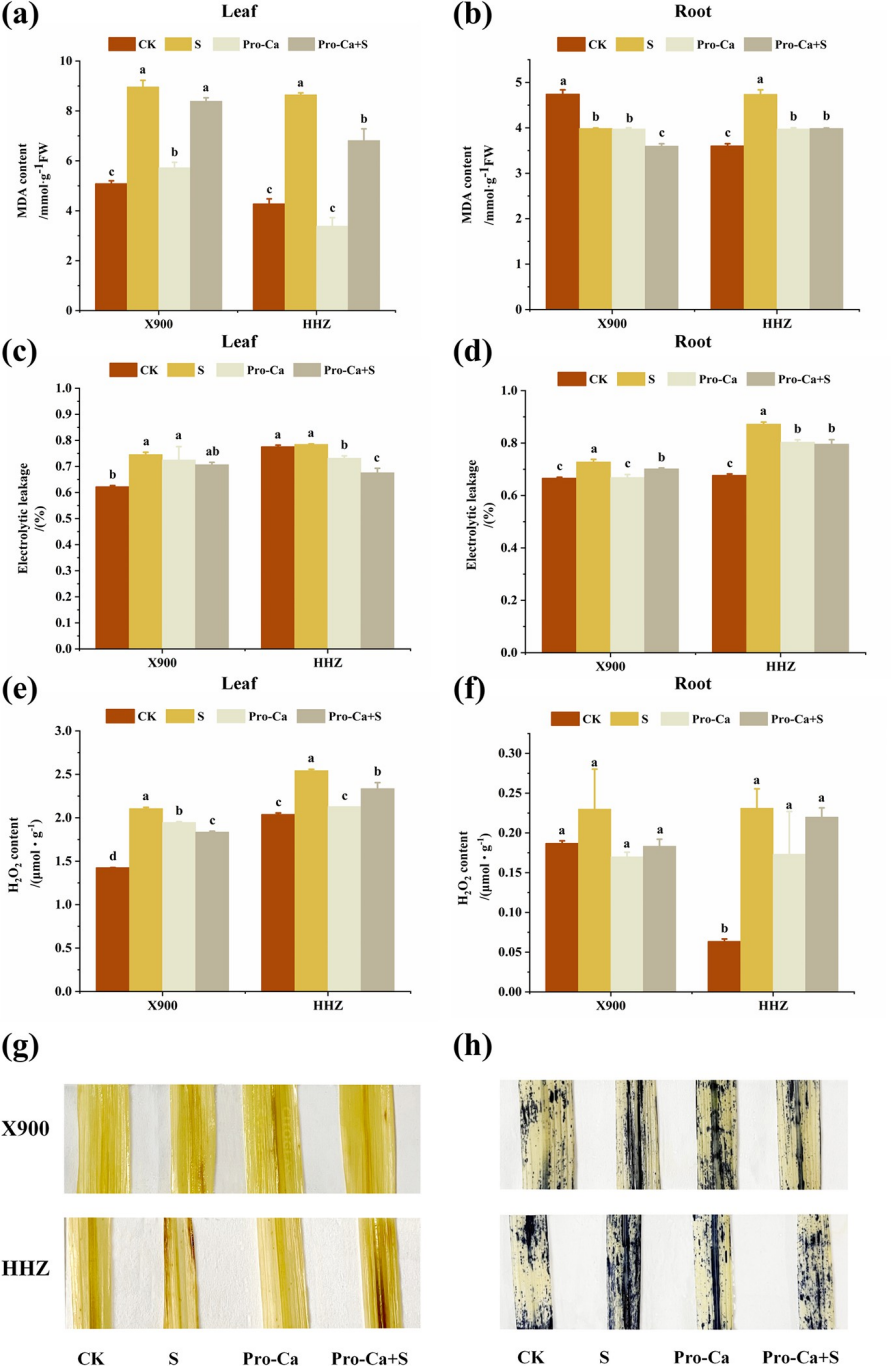
The alleviating effect of exogenous prohexadione-calcium (Pro-Ca) on the membrane damage and ROS accumulation of rice under salt stress. Malondialdehyde (MDA) content in the X900 (left) and HHZ (right) leaves (a) and roots (b), The electrolyte leakage (EL) in the X900 (left) and HHZ (right) leaves (c) and roots (d), The H_2_O_2_ content in the X900 (left) and HHZ (right) leaves (e) and roots (f), The H_2_O_2_ accumulation in the X900 and HHZ leaves (g) was determined by DBA staining, and the O_2_^∙−^ production rate in the X900 and HHZ leaves (h) was determined by NBT staining. Abbreviations: CK (water spraying + 0% NaCl), S (water spraying + 0.3% NaCl), Pro-Ca (100 mg∙L^-1^ Pro-Ca leaf spraying + 0.3% NaCl), and Pro-Ca+S (100 mg∙L^-1^ Pro-Ca leaf spraying + 0.3% NaCl). The numerical value represents the mean ± SE (*n =* 3), and different lowercase letters indicate significant differences according to Duncan’s test (*p <* 0.05).

The MDA content and electrolyte leakage rate are key indices that reflect the degree of membrane damage. Fig 4A and 4C show that under the NaCl stress, the MDA content in the leaves of the X900 and HHZ plants increased by 76.51% and 102.53%, respectively, and the electrolyte leakage rate increased by 19.98% and 1.25%, respectively. The MDA content in the roots of the X900 and HHZ plants increased by 31.68% and 81.61%, respectively, and the electrolyte leakage rate increased by 9.47% and 29.01%, respectively (Fig 4B and 4D). The salt stress aggravated the membrane lipid peroxidation of the rice leaves, increasing the H_2_O_2_ content and O_2_^∙−^ production rate in the leaves (Fig 4G and 4H). Under the salt stress conditions, the H_2_O_2_ content in the leaves and roots of the X900 and HHZ plants increased by 48.02% and 24.87%, 23.19%, and 265.09%, respectively, relative to the CK treatment group plants (Fig 4E and 4F).

The spraying of Pro-Ca significantly alleviated the membrane damage of the rice roots and leaves (Fig 4). Compared with the S treatment, the MDA content in the leaves of X900 and HHZ was improved by 14.86% and 41.90% in the Pro-Ca+S treatments, respectively, and the electrolyte leakage rate and MDA content in the roots of the two varieties were improved by 41.59% and 38.89% and 65.92%, and 15.99%, respectively (Fig 4A–4D). Spraying Pro-Ca also reduced the H_2_O_2_ content in the leaves of the two rice varieties by 39.51% and 40.78%, 107.85%, and 6.62%, respectively (Fig 4E and 4F). From the coloured figures, it can be seen that exogenous spraying of Pro-Ca reduces the content of H_2_O_2_ and the O_2_^∙−^ production rate in the rice leaves.

### The effects of exogenous prohexadione-calcium (Pro-Ca) on the antioxidant system of rice leaves and roots at the tillering stage under salt stress

**Fig 5:**
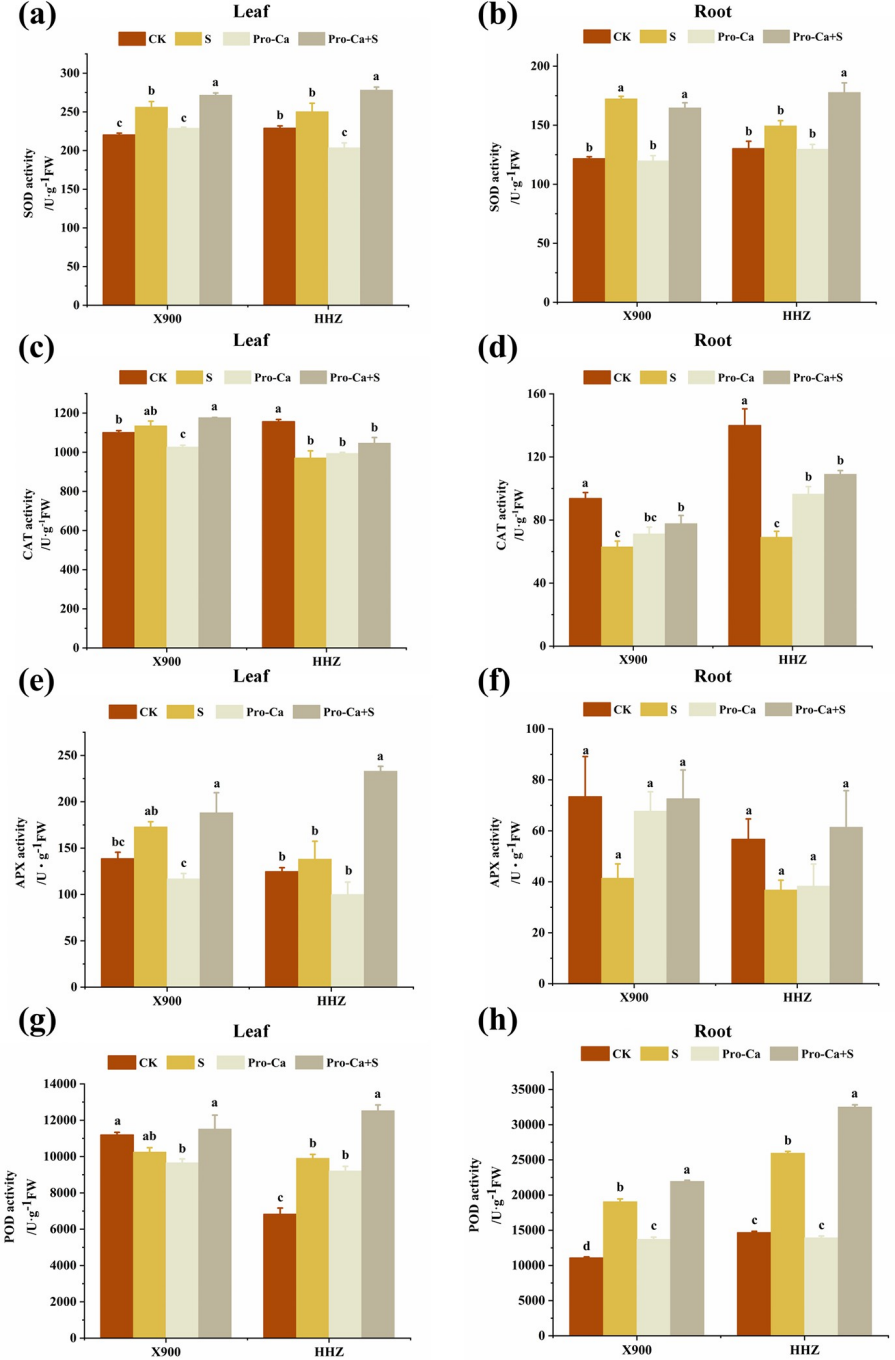
The alleviating effect of exogenous prohexadione-calcium (Pro-Ca) on the antioxidant enzyme activity of rice under salt stress. The superoxide dismutase (SOD) activity in the X900 (left) and HHZ (right) leaves (a) and roots (b); The katalase (CAT) activity in the X900 (left) and HHZ (right) leaves (c) and roots (d); The ascorbate peroxidase (APX) activity in the X900 (left) and HHZ (right) leaves (e) and roots (f); The peroxidase (POD) activity in the X900 (left) and HHZ (right) leaves (g) and roots (h). Abbreviations: CK (water spraying + 0% NaCl), S (water spraying + 0.3% NaCl), Pro-Ca (100 mg∙L^-1^ Pro-Ca leaf spraying + 0.3% NaCl), and Pro-Ca + S (100 mg∙L^-1^ Pro-Ca leaf spraying + 0.3% NaCl). The numerical value represents the mean ± SE (*n =* 3), and different lowercase letters indicate significant differences according to Duncan’s test (*p <* 0.05).

Under salt stress, the SOD activities in the leaves and roots of X900 and HHZ plants increased by 16.29% and 9.33%, 41.69%, and 14.83%, respectively, compared with the non-stress control condition (Fig 5A and 5B). Under salt stress, the CAT activity of the X900 leaves increased by 3.19%, that of the HHZ leaves decreased by 16.02%, and that of the roots of the two rice varieties decreased by 32.70% and 50.59%, respectively, compared with the control (Fig 5C and 5D). The POD activity of the leaves of X900 decreased by 8.41%, that of HHZ increased by 45.23%, and that of the roots of X900 and HHZ increased by 72.32% and 77.15%, respectively (Fig 5). The APX activity of the leaves of X900 and HHZ increased by 24.82% and 10.84%, respectively, while that of roots decreased by 35.01% and 43.50%, respectively, under the salt stress.

Compared with the S treatment group, spraying Pro-Ca increased the SOD activity in the leaves of the two rice varieties by 6.08% and 11.14%, respectively, and the CAT activity of the leaves of X900 and HHZ increased by 3.66% and 7.75%, respectively. The CAT activity of the roots increased by 23.34% and 57.71% in the X900 and HHZ plants, respectively, in the Pro-Ca+S treatment group. Spraying Pro-Ca increased the POD activity of the leaves and roots in the two rice varieties by 12.34% and 26.45%, 15.14%, and 25.27%, respectively, compared with the salt stress treatment. The application of Pro-Ca also increased the APX activity of the leaves of the two rice varieties by 8.82% and 68.73%, respectively, while that of the roots of X900 and HHZ increased by 75.26% and 66.81%, respectively (Fig 5).

**Fig 6:**
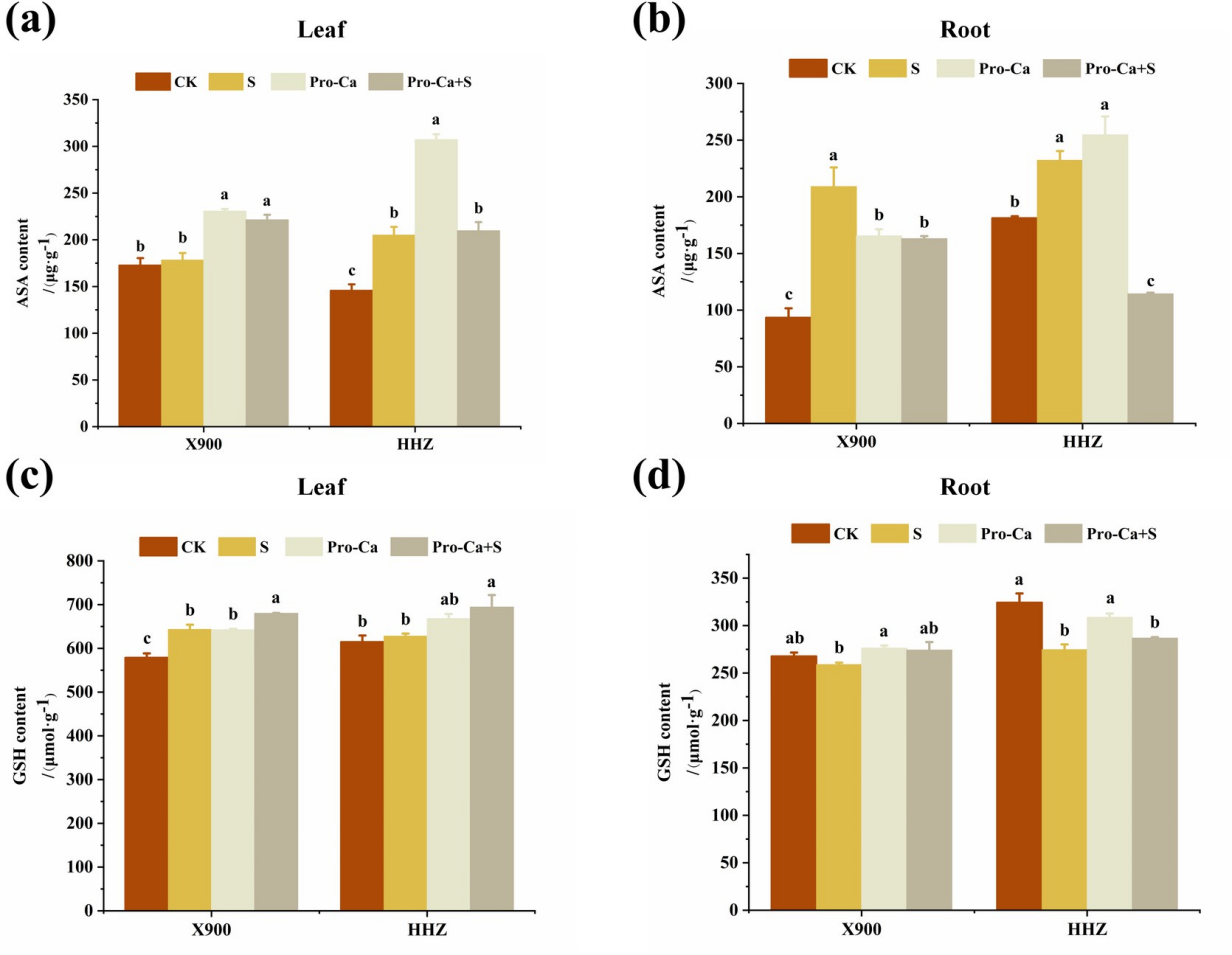
The alleviating effect of exogenous prohexadione-calcium (Pro-Ca) on the antioxidant enzyme activity of rice under salt stress. The ascorbate (AsA) content activity in the X900 (left) and HHZ (right) leaves (a) and roots (b); The glutathione (GSH) in the X900 (left) and HHZ (right) leaves (c) and roots (d). Abbreviations: CK (water spraying + 0% NaCl), S (water spraying + 0.3% NaCl), Pro-Ca (100 mg∙L^-1^ Pro-Ca leaf spraying + 0.3% NaCl), and Pro-Ca + S (100 mg∙L^-1^ Pro-Ca leaf spraying + 0.3% NaCl). The numerical value represents the mean ± SE (*n =* 3), and different lowercase letters indicate significant differences according to Duncan’s test (*p <* 0.05).

Under salt stress, the AsA activities of the leaves and roots of the X900 and HHZ plants were significantly increased by 40.76% and 3.31%, 28.08%, and 123.48%, respectively, and the GSH content of the leaves of X900 and HHZ increased by 2.15% and 11.13%, while that of the roots decreased by 15.35% and 3.33%, respectively, compared with the CK treatment group. Spraying Pro-Ca increased the AsA content of the two rice leaves but did not increase the AsA content of the roots. The GSH content in the roots and leaves of the two rice varieties sprayed with Pro-Ca increased significantly (Fig 6).

### The effects of exogenous prohexadione-calcium (Pro-Ca) on the osmotic adjustment substances in rice leaves and roots at the tillering stage under salt stress

**Fig 7:**
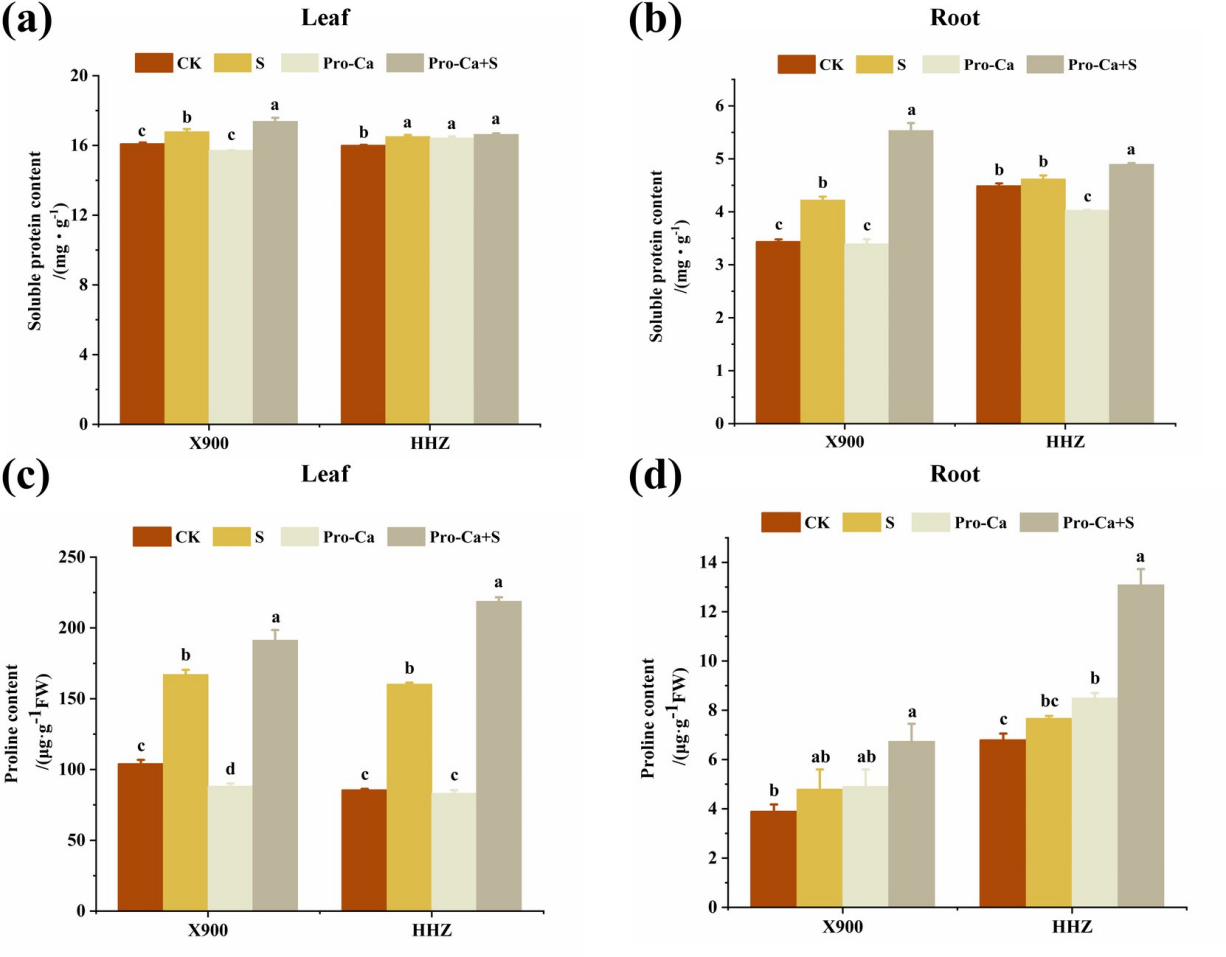
The alleviating effect of exogenous prohexadione-calcium (Pro-Ca) on the antioxidant enzyme activity of rice under salt stress. The soluble protein content in the X900 (left) and HHZ (right) leaves (a) and roots (b); the proline content in the X900 (left) and HHZ (right) leaves (c) and roots (d). Abbreviations: CK (water spraying + 0% NaCl), S (water spraying + 0.3% NaCl), Pro-Ca (100 mg∙L^-1^ Pro-Ca leaf spraying + 0.3% NaCl), and Pro-Ca + S (100 mg∙L^-1^ Pro-Ca leaf spraying + 0.3% NaCl). The numerical value represents the mean ± SE (*n =* 3), and different lowercase letters indicate significant differences according to Duncan’s test (*p <* 0.05).

Under salt stress, the contents of osmotic adjustment substances in the two rice varieties increased significantly, among which the soluble protein content of the leaves and roots of X900 increased by 4.39% and 22.95%, respectively, while that of HHZ increased by 3.24% and 2.99%, respectively, compared with CK treatment group. The proline content of the leaves and roots of X900 and HHZ increased by 60.80% and 87.67%, 23.48% and 13.08%, respectively (Fig 7).

After externally spraying Pro-Ca, the soluble protein content of the leaves and roots of the X900 and HHZ plants increased by 3.54% and 0.07%, 31.04% and 5.97%, and the proline content increased by 14.48% and 36.48%, 40.34% and 70.53%, respectively (Fig 7).

### Correlation analysis

**Fig 8:**
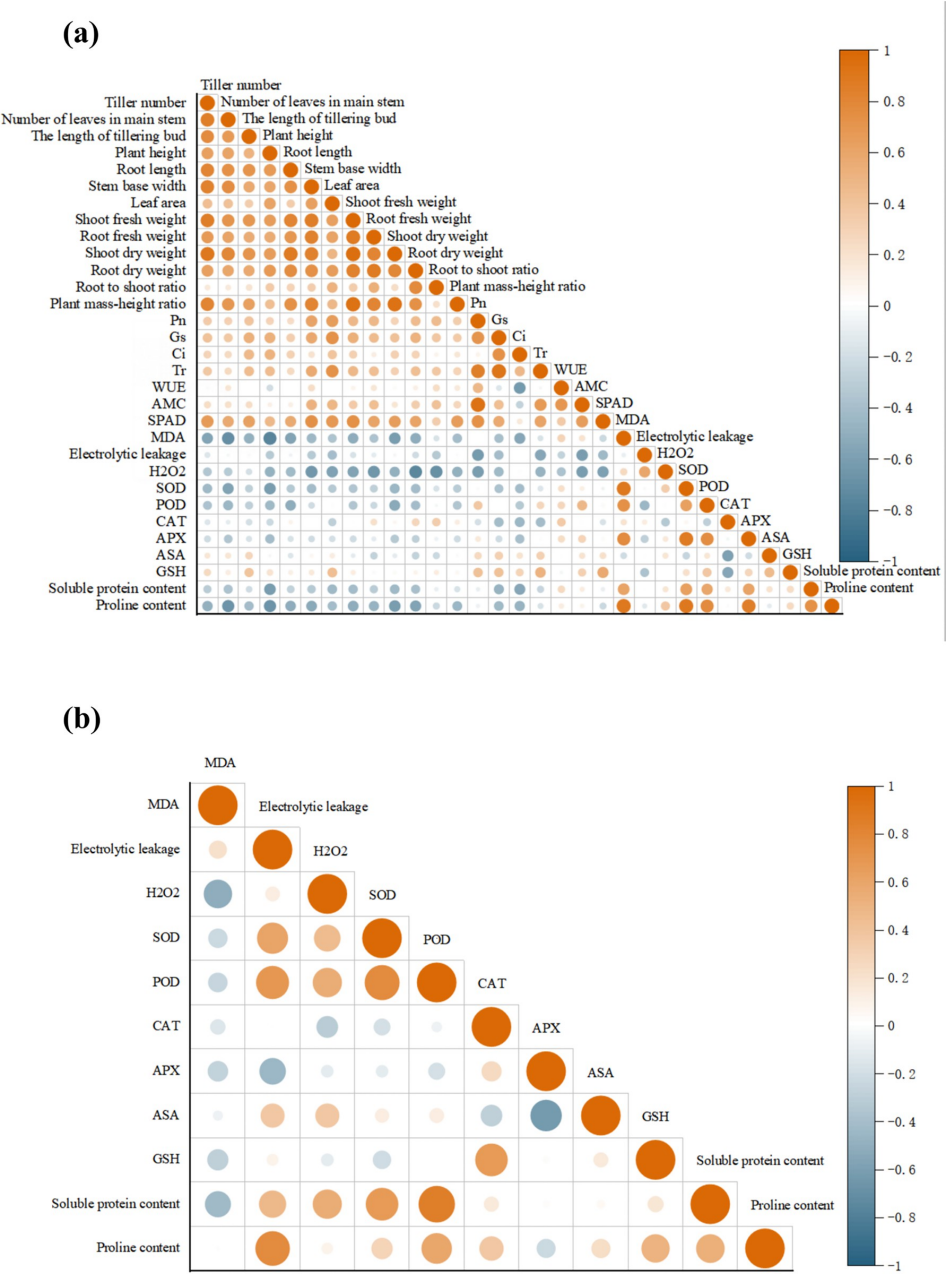
The correlation analysis of tillering ability, photosynthesis, and antioxidant indices in different parts of the rice. The correlation analysis of the rice leaves (a) and roots (b). Significant difference at the level of *P <* 0.05.

To study the relationship between the tillering ability, ROS metabolism and the antioxidant capacity of the rice at the tillering stage in the different treatment groups, a correlation analysis of morphological and physiological indices of the leaves and roots of the two rice varieties was carried out. As shown in Fig 8, the aboveground biomass of the rice leaves at the tillering stage was positively correlated with Pn, Gs, Ci, and Tr, with a weaker association. Meanwhile, the correlation coefficient was distributed between 0.26 and 0.45. SPAD was positively correlated with Pn, Gs, and Tr, with correlation coefficients ranging from 0.47 to 0.75. The MDA content, electrolyte leakage rate, and H_2_O_2_ content were negatively correlated with Pn, Gs, Ci, and Tr. SOD was positively correlated with POD and APX, with correlation coefficients of 0.68 and 0.82, respectively. SOD, POD, and APX are positively correlated with soluble protein and proline. Fig 8B shows that the MDA content in the rice roots was negatively correlated with the activities of SOD, POD, CAT, and APX, with correlation coefficients ranging from -0.15 to -0.27. The soluble protein content in the roots was positively correlated with SOD and POD, and the correlation coefficients were 0.68 and 0.85, respectively. Through correlation analysis, we found that the salt tolerance of rice was positively correlated with photosynthetic capacity, ROS metabolism, antioxidant indices, and osmotic adjustment substances, indicating that all physiological indices might be involved in the regulatory mechanism of salt tolerance in rice (Fig 8).

## Discussion

### Morphological indices

Morphological indicators can directly reflect the effects of salt stress and abiotic stress on plant growth [37]. Studies have shown that 100 mM NaCl stress can significantly reduce plant height, stem base width, leaf area, fresh weight, and dry weight of the aboveground and belowground parts of eggplant seedlings [38]. Similar results were found in wheat seedlings and rice [39]. Consistent with the previous research results, in this experiment, salt stress reduced the tillering number, the number of main stems and leaves, the length of tillering buds, plant height, root length, stem base width, leaf area, and the aboveground and belowground biomass of the two rice varieties at the tillering stage. These results indicated that salt stress inhibited the growth of rice at the tillering stage. Compared with the S treatment group, the length of the tiller bud, the number of tillers, the width of the stem base, the length of the root, the aboveground and belowground biomass, and other morphological indices of the two rice varieties treated with Pro-Ca increased. Khan et al. [40] showed that exogenous Pro-Ca improved the lodging resistance of flax by increasing the base width of the flax stem. Iqbal and Ashraf [41] also confirmed that GA3 effectively alleviated the effects of salt stress on plant height, tiller number, and the aboveground biomass of wheat. The results indicated that Pro-Ca could effectively alleviate the inhibition of salt stress on growth indices in the rice tillering stage. Kim et al. [42] also showed that Pro-Ca delayed senescence by inhibiting ethylene production in plants. At the same time, we found that Pro-Ca inhibited the plant height of rice at the tillering stage but increased the number of main stems and leaves compared with the salt stress treatment. This may be because the regulatory effect of GA on cells is mainly reflected in cell elongation rather than cell division.

### Photosynthetic capacity

Photosynthesis is an essential physiological process of plants, and photosynthetic indicators can effectively reflect the growth of plants [43]. Salt stress reduces the contents of Pn, Tr, Gs, WUE, chlorophyll, and the activity of photosystem II (PSII) in plants, and with the aggravation of soil salt stress, the photosynthetic capacity gradually decreases [44–46]. The chlorophyll content is also an important indicator of plant tolerance to abiotic stress [47]. In this experiment, salt stress reduced the Pn, Tr, Ci, and Gs of the two rice varieties at the tillering stage, which indicated that salt stress damaged the photosynthetic capacity of the rice at the tillering stage. The simultaneous decrease in Ci and Gs indicated that the decrease in the photosynthetic rate in this experiment was caused by stomatal factors [45]. In this experiment, the photosynthetic capacity and SPAD value of the two rice varieties sprayed with exogenous Pro-Ca under salt stress were significantly higher than those treated with salt stress alone. This shows that Pro-Ca alleviated the damage of salt stress to the photosynthetic capacity of the rice leaves at the tillering stage, and previous studies have reached similar conclusions. Applying exogenous Pro-Ca increased the Pn of plants by increasing the chlorophyll content of apple and pear leaves [48]. Exogenous Pro-Ca has also been shown to increase chlorophyll content in onion and cucumber leaves [49, 50]. At the same time, we found that under salt stress, the Ci of the two rice varieties sprayed with exogenous Pro-Ca decreased, which indicated that Gs might not be the only factor of the Pro-Ca-induced photosynthesis changes under salt stress [51].

### Membrane damage index and H_2_O_2_ content

The MDA content and electrolyte leakage rate are important indicators of the degree of lipid peroxidation, which represents the degree of cell membrane damage [52–54]. Tang et al. [47] showed that salt stress increases the MDA content and O_2_^·-^ production rate in cucumber leaves. Yan [55] showed that salt stress also increased the MDA content and electrolyte leakage rate in maize leaves. Hasanuzzaman et al. [56] made a similar conclusion for Brassica napus. In this study, salt stress significantly increased the H_2_O_2_ content, electrolyte leakage rate, and the MDA content in the leaves and roots of the two varieties, indicating that salt stress may produce excessive ROS due to the imbalance of electron transfer, resulting in an increase in the H_2_O_2_ content in rice at the tillering stage. And thus, resulting in the oxidative damage to the cell membrane and an increase in the MDA content and electrolyte leakage rates [53]. Compared with the salt treatment alone, the H_2_O_2_ content, MDA content, and electrolyte leakage rate in the leaves and roots treated by spraying Pro-Ca before the salt stress were significantly reduced. Soleimani [24] also obtained similar research results on tomatoes, which indicated that exogenous spraying of Pro-Ca reduced the production of ROS in the rice tillering stage and the oxidative damage caused by salt stress.

### Antioxidant system

Abiotic stress will cause oxidative stress to plant cells, which will cause them to produce more ROS in the process of photosynthesis and metabolism [51, 57]. To improve their viability, plants themselves will respond to salt stress by regulating salt tolerance mechanisms [58–60]. This experiment found that under salt stress, the SOD activity in the roots and leaves of the two varieties and the APX activity in the leaves of the two varieties increased significantly. The results showed that salt stress could activate the antioxidant system in rice at the tillering stage and improve the stress resistance of rice. Farhangi-Abriz and Torabian [61] found that under NaCl stress (6 and 12 dS/m), the activities of the antioxidant enzymes in the leaves and roots of kidney beans increased. Salt stress also increased antioxidant enzyme activity in tomato leaves [62]. Notably, in our study, salt stress significantly increased the CAT activity in the leaves of the X900 plants but decreased the CAT activity in the leaves and roots of the HHZ plants and decreased the APX activity in the roots of the two varieties. Noreen and Ashraf [63] found that salt stress can increase the activities of SOD and POD in pea leaves, but a higher salt level will lead to a decrease in CAT activity in pea varieties, which is similar to the results of this study. Antioxidants such as AsA, GSH, and polyphenols play an important role in the stress response of plants. In this study, salt stress significantly increased the AsA content in the leaves and roots of the two varieties and increased the GSH content in the leaves but decreased the GSH content in the roots. Previous studies have drawn similar conclusions about the various dynamics of AsA and GSH content levels in plants under salt stress. Ding et al. showed that salt stress increased GSH and AsA content levels in wheat leaves [64]. Zhang et al. [65] showed that compared with the control, salt stress also increased the contents of AsA, DHA, and GSH in cucumber seedling leaves. However, Nahar et al. [66] showed that salt stress significantly reduced the contents of AsA and GSH in mung bean seedlings. In our study, after spraying Pro-Ca, the antioxidant enzyme activities (SOD, POD, APX, and CAT) of the rice roots and leaves under salt stress were significantly higher than those under salt stress alone. Previous studies have also confirmed that Pro-Ca can promote oxidase in cucumbers and soybean [23, 67]. Under salt stress, the contents of GSH and AsA in the rice roots and leaves sprayed with Pro-Ca increased significantly in our study. However, there was no significant effect on the AsA content in the roots, which indicated that exogenous Pro-Ca might be involved in regulating the ROS scavenging system under salt stress, thus improving the antioxidant capacity of rice.

### Osmotic adjustment substances

Osmotic substances protect the average growth and metabolism of plants by maintaining cell expansion pressure [68]. The contents of osmotic adjustment substances such as proline and soluble protein can be used as physiological indices of plant stress resistance [53, 69]. In this experiment, salt stress significantly increased the content of osmotic adjustment substances (soluble protein and proline) in the roots and leaves of the two rice varieties at the tillering stage. Consistent with previous research results, Gandonou et al. [69] showed in previous experiments that salt stress significantly increased the contents of proline and soluble sugar in sugarcane leaves and roots, resulting in an increase in soluble protein concentration in the leaves and roots of salt-tolerant varieties and a decrease in soluble protein concentration in the leaves and roots of sensitive varieties. In addition, under 120 mM NaCl stress, the contents of proline, soluble sugar, and soluble protein in wheat increased significantly. Proline can keep water in the cytoplasm, and the increase in its content indicates that plants may have better tolerance to stress [43, 70–72]. In our study, exogenous spraying of Pro-Ca significantly increased the contents of proline and soluble protein in the roots and leaves of the two rice varieties at the tillering stage. Previous studies also reached a similar conclusion. Soleimani [24] treated tomatoes with Pro-Ca, which increased the proline content in the tomato fruits. Bekheta et al. [22] reported that the application of Pro-Ca increased the proline content in broad bean seedlings under salt stress. This shows that Pro-Ca can detoxify ROS by promoting the synthesis of soluble protein and proline content, protecting the integrity of the cell membrane and the stability of antioxidant enzymes, thus protecting the growth and development of plants [73].

**Fig 9:**
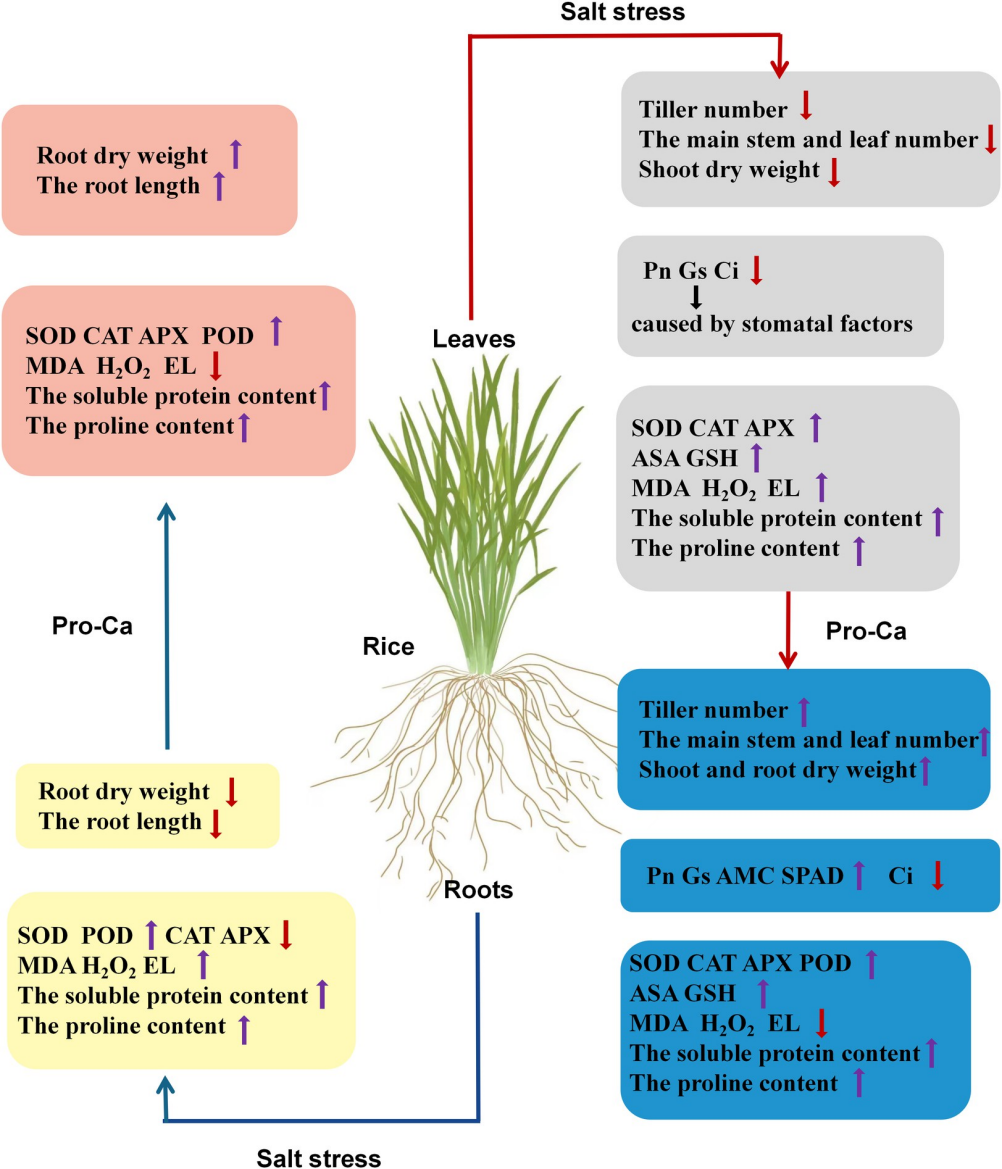
A proposed model shows the damage of salt stress to rice tillering and the regulatory effect of Pro-Ca, with the upwards arrow indicating a promotion effect and the downwards arrow indicating an inhibition effect.

In summary, Pro-Ca alleviated the inhibition of salt stress on the rice plant’s tillering ability. Exogenous application of Pro-Ca increased the tillering number, the number of main stems and leaves, the length of tillering buds, the chlorophyll content, the photosynthetic capacity of the rice leaves, the antioxidant capacity, and the osmotic adjustment capacity of the rice leaves and roots at the tillering stage and effectively improved the salt tolerance and quality of the rice at the tillering stage (Fig 9).

## Conclusion

In summary, salt stress harmed photosynthesis, membrane lipid peroxidation, osmotic adjustment ability, and the antioxidant system of the rice at the tillering stage, while the application of exogenous Pro-Ca effectively alleviated the toxicity of the salt stress and improved the phenotypic characteristics of the rice, such as tillering number and number of leaves in the main stem. We hypothesize that exogenous Pro-Ca alleviated the salt stress damage to the rice tillering stage by improving the photosynthetic capacity, antioxidant system, and the osmotic adjustment capacity. These results indicate that foliar spraying of Pro-Ca may be an effective method to improve the salt tolerance of rice and provide a reference for further research on the specific regulatory mechanism of Pro-Ca to improve the salt tolerance of rice at the tillering stage.

## Supporting information

S1 Data (XLSX)
